# A systematic review investigating interventions that can help reduce consumption of sugar-sweetened beverages in children leading to changes in body fatness

**DOI:** 10.1111/jhn.12267

**Published:** 2014-09-19

**Authors:** A Avery, L Bostock, F McCullough

**Affiliations:** Division of Nutritional Sciences, University of NottinghamLeics, UK

**Keywords:** childhood obesity, sugar sweetened beverages, dietary intervention, public health, systematic review

## Abstract

**Background:**

Both the prevalence of childhood obesity and the consumption of sugar-sweetened beverages (SSBs) have increased globally. The present review describes interventions that reduce the consumption of SSBs in children and determines whether this leads to subsequent changes in body fatness.

**Methods:**

Three databases were searched from 2000 to August 2013. Only intervention control trials, ≥6 months in duration, which aimed to reduce the consumption of SSBs in >100 children aged 2–18 years, and reporting changes in body fatness, were included. The quality of selected papers was assessed.

**Results:**

Eight studies met inclusion criteria. Six interventions achieved significant (*P* < 0.05) reductions in SSB intake, although this was not always sustained. In the two interventions providing replacement drinks, significant differences in body mass index (12- or 18-month follow-up) were reported (*P* = 0.001 and 0.045). The risk of being overweight/obesity was reduced (*P* < 0.05) in three of the five education programmes but in one programme only for girls who were overweight at baseline and in one programme only for pupils perceived to be at greater risk at baseline. In the one study that included both provision of water and education, the risk of being overweight was reduced by 31% (*P* = 0.04) in the intervention group.

**Conclusions:**

The evidence suggests that school-based education programmes focusing on reducing SSB consumption, but including follow-up modules, offer opportunities for implementing effective, sustainable interventions. Peer support and changing the school environment (e.g. providing water or replacement drinks) to support educational programmes could improve their effectiveness. Home delivery of more suitable drinks has a big impact on reducing SSB consumption, with associated reductions in body weight.

## Introduction

Childhood obesity is a major public health problem in many countries across the world. Dietary interventions that could prevent excess weight gain in childhood are required.

There has been a growing trend globally in the consumption of sugar-sweetened beverages (SSBs) amongst children. A recent large US study of trend and cross-sectional analyses (Han & Powell, 2013) drawn from National Health and Nutrition Examination Survey (NHANES) data between 1999–2008 showed that the prevalence of heavy total SSB consumption ≥ 2092 kJ day^−1^ [≥ 500 kcal day^−1^] had increased amongst children (4–5%). Low-income children had higher odds [odds ratio = 1.93; 95% confidence interval (CI) = 1.05–3.56] of heavy consumption than high-income ones, thus highlighting a potential targeting strategy for public health interventions.

A large cross-sectional study of European adolescents (Duffey *et al*., 2012) highlighted how SSBs provide more daily energy intake (30.4% of total beverage intake) than any other beverages. Amongst British children, energy from drinks accounts for 14% of energy intake in children aged between 4 and 18 years, with sugary drinks accounting for the bulk of that energy and SSB intake being particularly high amongst adolescents (Ng *et al*., 2012). Although a comparison between 1983 and 1997 SSB intake by UK children aged 10–11 years and 14–15 years showed a significant increase, this did not reflect in an increase in total sugar intake (115 g day^–1^ in 1983 and 113 g day^–1^ in 1997) (Gibson, 2010).

There is ongoing current debate around whether there is sufficient scientific evidence that decreasing consumption of sugar-sweetened beverages (SSBs) reduces obesity (Hu, 2013; Kaiser *et al*., 2013). The World Health Organization recently carried out a meta-analysis of cohort studies in children which found that a higher intake of SSBs was associated with a 55% (95% CI = 32–82%) higher risk of being overweight or obese (Morenga *et al*., 2013).

Putting this debate to one side, SSBs are nutrient poor and provide ‘empty’ energy to children's diets.

This systematic review aims to clarify which interventions aimed at children help to reduce the consumption of SSBs and whether these interventions lead to subsequent changes in body fatness. The methods of delivery of these interventions will be considered to help practitioners plan future interventions in this area.

For the purposes of the present review, the definition of SSBs includes carbonated and noncarbonated drinks sweetened with sugar, including fruit juices and milk drinks.

## Materials and methods

The systematic review followed the relevant criteria of the PRISMA statement (Preferred Reporting Items for Systematic reviews and Meta-Analysis) (Liberati *et al*., 2009).

### Study selection

Trials, published in English from 2000 onwards, were included in this review if they met certain criteria: (i) the trial involves ≥100 healthy children, including healthy weight, overweight and obese participants, aged between 2–18 years; (ii) the intervention includes a focus on reducing consumption of sugary drinks; (iii) control data are available for comparison; (iv) the results report on the change in consumption of SSBs AND changes in body fatness; and (v) the study comprises an intervention study period of ≥6 months in duration.

The primary outcome measure of interest was the reduction in consumption of sugary drinks compared to control data. The secondary outcome measure was any change in body composition indicative of body fatness [e.g. change in body mass index (BMI) from baseline, percentage overweight or obese, risk of being overweight, skinfold thickness or waist circumference].

To be included in the review, any multi-component education programme needed to list the reduction of SSBs as the number one priority.

### Search strategy

Searches were undertaken using the following electronic databases: Web of Science, Medline and EMBASE. The search centred on identifying intervention studies amongst populations of children (aged between 2–18 years) that reduced their consumption of SSBs from January 2000 to August 2013. Two searches were undertaken. First, the search terms used were [fruit juice or soft drink or sugar-sweetened beverage or nutritively sweetened beverage or soda or liquid calories or chocolate milk] AND [children or kids or adolescents] AND [energy]. A further search was undertaken using [sugar free drink or diet drinks or diet sodas] AND [children or kids or adolescents] AND [energy].

### Data collection and extraction

**Figure 1:**
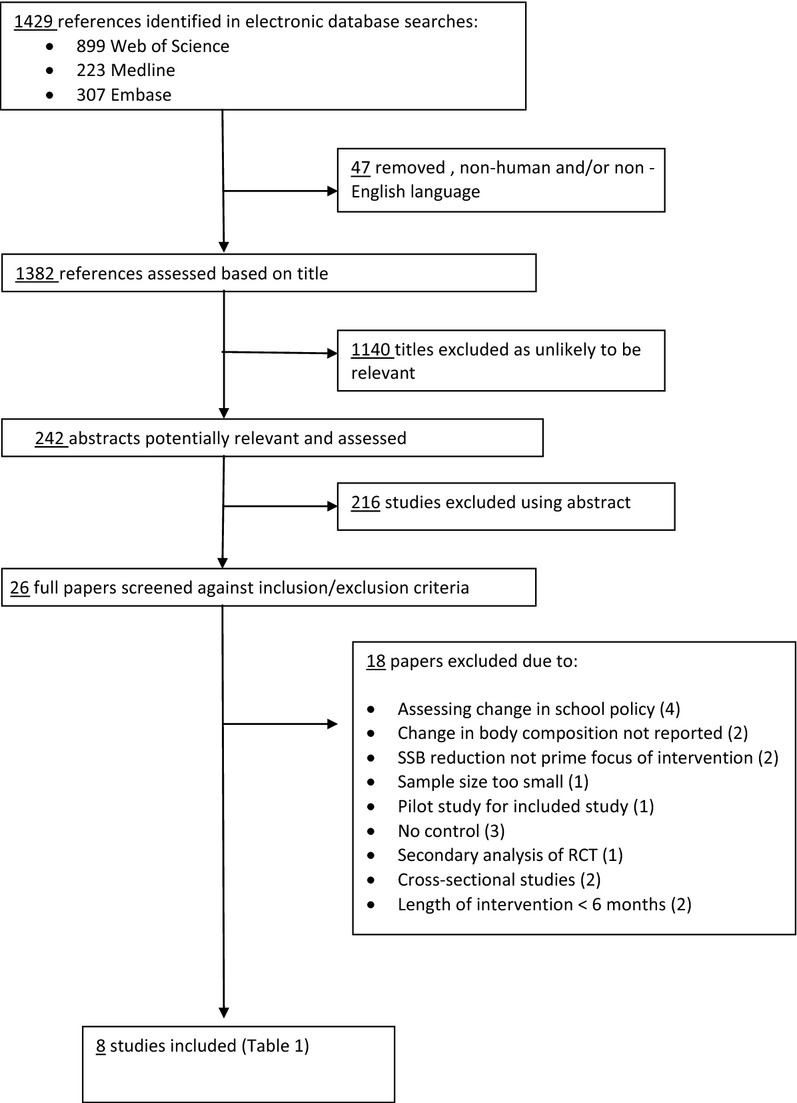
Flow diagram for database search results. RCT, randomised controlled trial; SSB, sugar-sweetened beverage.

Titles and abstracts of studies were identified by the database searches using the search strategy outlined. Nonhuman trials and non-English language studies were eliminated at this stage using database filters. Studies were then screened against the inclusion criteria using title screening, abstract screening and full paper screening. Title screening was carried out by one reviewer, with a second reviewer consulted at the abstract screening stage, and a third reviewer in the full paper screening process. The screening process, including the number of studies excluded at each stage in the process, was documented and is shown in Fig.1.

Data from each of the final eight studies identified by the search process was extracted using a data extraction form adapted for this systematic review. The headings used in the form include: author, country of origin, number of subjects, mean age, weight status of subjects, where study is based, length of intervention and any follow-ups, details of the intervention, change in consumption of SSBs, change in body fatness compared to control group and overall study conclusion.

For ease of comparison of changes in SSB consumption between studies, raw data from studies were converted into percentage change from baseline for both the intervention and control arms where possible.

### Quality assessment

The quality of the final eight studies was critically assessed using the Jadad scale for reporting randomised controlled trials (Jadad *et al*., 1996). The quality scores ranged between 1 and 5 with higher scores indicating higher study quality.

## Results

### Interventions targeting the consumption of sugar-sweetened beverages

The present review identified eight studies which met the strict inclusion criteria (Fig.1) and included interventions focused on reducing the consumption of SSBs with data on changes in body fatness reported (Table1). A follow-up study to James *et al*. (2004) was carried out by the same group in 2007 and the results of these studies have been considered and reported together.

**Table 1:** Controlled studies with interventions that can lead to reduced consumption of SSBs leading to changes in body fatness

First author (year), country	Total number of subjects, mean age, weight status of subjects, where study based	Length of intervention (months), timing of follow-up (months)	Intervention	Change in consumption from sugary drinks (intervention versus control)			Change in BMI (kg m^–2^ (intervention versus control)	Overall study conclusion
Cunha (2013), Brazil (area of high social deprivation)	559, 11, Normal, school	9	9 nutritional education sessions delivered by trained nutritionists	Variation in daily frequency:			No difference between intervention and control groups	Encouraging adoption of healthy eating promoted a reduction in SSBs but did not lead to BMI reduction. Possible substitution of SSBs with sugar-containing juices
					Intervention	Control		
				Sodas (*P* = 0.02)	−0.2	−0.08		
				Juices (*P* = 0.66)	−0.16	0.01		
De Ruyter (2012), Netherlands	641, 8, Normal, school	18	Provision of masked (non-energy and sugar-sweetened) canned drinks daily whilst in school	Mean (SD) baseline consumption of 1.02 (0.20) during school break and 1.50 (1.40_ per weekend day. [Each sugar-sweetened drink provided 435KJ/104kcal]			Change in BMI *Z*-score; intervention 0.02. Control group 0.15 (*P* = 0.001) Weight increase; 6.35 versus 7.37 kg	Masked replacement of sugar-containing beverages with non-energy beverages reduced weight gain and fat accumulation in normal weight children
Ebbeling (2012), United States	224, 15, Overweight and obese, home	12, 24	2 weekly home delivery of non-energy beverages with written intervention messages, monthly telephone calls with parents, three check-in visits with participants	Servings day^–1^ (*P* < 0.001): *At 12 months – 88% decrease* *At 24 months – 76% decrease* kJ day^−1^ (*P* < 0.001): *At 12 months – 88% decrease* At 24 months – 79% decrease			12 months: Significant effect of −0.57 (*P* = 0.045) *24 months:* Nonsignificant effect of –0.30 (*P* = 0.46)	In overweight and obese adolescents, the increase in BMI was smaller in the intervention group after 1 year but not at 2-year follow-up
Ezendam (2012), Netherlands	883, 13, Normal, Web-based and school	4, 24	8 computer modules on weight management and energy balance-related behaviours	Lower odds (0.54) of consuming >400 mL day^−1^ of SSB in intervention group. No significant differences at 24-month follow-up			No difference in BMI or waist circumference at 4 months or 24 months Those perceived to be at greater risk at baseline show differences in BMI change of 1.5 versus 3.7	Intervention associated with positive short-term effects on SSB consumption but a reduced BMI only in those children perceived to be at greater risk at baseline
James (2004, 2007), England	644, 9, Normal, school	12, 36	Nutritional education programme, ‘Ditch The Fizz’, delivered (by research investigator) through school. 1 × 1-h session each term (four sessions in total)	No of glasses/3 days: 32% (*P* = 0.02) reduction in carbonated drinks. Nonsignificant reduction in carbonated drinks with sugar of 25% (*P* = 0.2) Change in consumption at 3-year follow-up not recorded			% overweight/obese: 12 months: Intervention –0.2% decrease Control – 7.5% increase 36 months: No difference between intervention and control	A targeted, school-based education programme produced a modest reduction in the number of carbonated drinks consumed that was associated with a reduction in the number of overweight and obese children Difference in overweight prevalence no longer seen at 3 years
Muckelbauer (2009), Germany	2950, 8, Normal, school	11	Water fountains and water bottles provided in schools plus educational programme of 4 × 45-min lessons on water delivered by teachers via the curriculum	Number of glasses day^–1^: Soft drinks – No significant effect (*P* = 0.406) [Juice – No significant effect (*P* = 0.500)] Water – 1.1 (*P* < 0.001)			Change in BMI SD score did not differ between groups Risk of being overweight was reduced by 31% (*P* = 0.04) in the intervention group	An environmental and educational, school-based intervention proved effective in the prevention of overweight among children in elementary school, even in populations from socially deprived areas
Sichieri (2008), Brazil	1140, 11, Normal, school	7	Healthy lifestyle education programme (10 × 1-h sessions delivered by research assistants) emphasising water consumption instead of sugar-sweetened carbonated beverages	Mean daily intake (mL day^–1^) Intervention group: Carbonated drinks – 23% decrease (*P* = 0.03) Juice – Nonsignificant increase of 3% (*P* = 0.08) Control group: Carbonated drinks – 4% decrease (*P* = 0.03) [Juice – Nonsignificant increase of 12% (*P* = 0.08)]			Not statistically significant between groups. Among those overweight at baseline, a reduction in BMI in the intervention group, statistically significant among girls	Decreasing SSBs intake significantly reduced BMI among overweight girls Efforts to reduce energy intake through liquids need to emphasise overall sweetened beverages and juices
Singh (2009) Netherlands	1108, 13, Normal, School	8, 12 and 20	School-based, multi component, health promotion, 11 lessons	mL day^–1^ (*P*-value not reported) *8 months* *Girls: 23.9% decrease* *Boys: 25.5% decrease* *12 months* *Girls: 26% decrease* *Boys: 20.7% decrease* No sig differences at 20 months			No significant differences in BMI between groups. (Significant difference in sum of skinfold thickness in girls at 8 and 12 months)	Beneficial effects on sum of skinfold thickness in girls and consumption of SSBs in girls and boys in short and long term

BMI, body mass index; SSB, sugar-sweetened beverage.

Of the eight studies selected, five were undertaken in Europe (one in England, one in Germany, three in the Netherlands), one in the USA, and two in Brazil. For one of the studies based in Brazil, schools from a particularly poor region were targeted for the intervention. Seven of the studies involved ‘normal’ weight children with one undertaken amongst overweight and obese children. Seven of the studies were conducted through schools, whereas one was home based. The children in the studies ranged in mean age between 8 and 15 years. The length of the interventions ranged between 4 and 18 months and the time of follow-up between 7 and 36 months, with a follow-up conducted at the end of the intervention in four of the studies. Sample sizes ranged from 224 to 2950 children.

No studies were identified amongst children aged less than 8 years.

These interventions can be considered under four headings: school-based educational programmes, school-based educational programme combined with environmental change, school-distributed drinks and home-delivered drinks.

### School-based educational programmes

The primary school-based educational intervention (‘Ditch the Fizz’ delivered as part of CHOPPS, the Christchurch Obesity Prevention Programme in Schools), undertaken by James *et al*. (2004), resulted in a 25% (*P* = 0.2) reduction in the number of glasses of carbonated drinks containing sugar consumed over a 3-day period in the intervention group, compared to the control group, and reported a mean difference of 7.7% (95% CI = 2.2–13.1%) in the percentage of being overweight or obese following the intervention at 12-month follow-up. Although there was an increase of 7.5% in the number of children who were overweight or obese in the control group, there was a 0.2% decrease in the intervention group over the initial 12-month follow-up period.

In terms of limitations, it is highlighted that the data on drink consumption was obtained for a short period (3 days) and relied on self-reported diaries, which may have been subject to under-reporting (Campbell *et al*., 2001). Furthermore, only 36% of baseline and follow-up drink diaries were returned. Two years after the end of the intervention, a follow-up study (James *et al*., 2007) found no difference in childhood overweight or obesity between the intervention and control group.

The school-based, healthy lifestyle education programme by Sichieri *et al*. (2008) encouraged water consumption in place of SSBs and resulted in a significant decrease of 23% (*P* = 0.03) in the mean daily intake (mL day^–1^) of carbonated drinks in the intervention group. This decrease was four times as big in the intervention group compared to the control group. There was no significant difference in BMI between the intervention and control groups but, amongst intervention girls who were overweight at baseline, a significant (*P* = 0.009) reduction in BMI was found at the end of the school year. It should be noted that, during the intervention, subjects reported increasing their consumption of fruit juices, which may have compensated for any energy reduction from SSB reduction. This led to the conclusion that any efforts to reduce energy consumption through liquids should emphasise overall sweetened beverages including sugar added juices rather than just sodas. It was also concluded that the switch from sugary drinks to plain water was potentially too large and that, in future interventions, a switch from high to low-sugar beverages might be more successful. One limitation of this study was that data was based on only one self-reported 24-h recall at baseline and a further 24-h recall after the intervention. However, an attempt was made to validate the baseline data by using an additional beverage frequency questionnaire.

Singh *et al*. (2009) found that SSB consumption in both boys and girls of secondary school age had significantly decreased (25.5% and 23.9%, respectively; *P*-value not reported) by the end of this 8-month, educational and environmental, health promotion intervention, and that this was largely maintained at 12 months but had disappeared by 20 months. By the end of the intervention, there was no significant difference in BMI between intervention and control groups, although the sum of skinfold thickness was lower in girls in the intervention group at 8 months (−2.3 mm; 95% CI = −4.3 to −0.3 mm) and 20 months (−2.0 mm; 95% CI = −3.9 to −0.1 mm). It is worth noting that the limitations of the present study include nonblinding of key research assistants, self-selection of participating schools and the use of self-report questionnaires.

Cunha *et al*. (2013) delivered nine nutritional education sessions by trained nutritionists as part of their PAPPAS (‘parents, students and teachers for healthy eating’) intervention. Parents and teachers received information on the same subjects, the positive messages related to the intake of water, fruits, rice and beans and an emphasis on reducing SSBs and cookies.

The 9-month intervention led to a significant variation in the daily frequency of consumption of SSBs, specifically sodas (−0.2 in the intervention group compared to −0.08 in the control; *P* = 0.02) amongst the 11-year-olds but did not result in a reduction in BMI gain. However, the two groups were not well matched at baseline, with a higher prevalence of being overweight and obesity and a slightly higher intake of SSBs amongst participants in the control group. Cunha *et al*. (2013) commented on the additional difficulties of trying to make changes in low socioeconomic groups in developing countries such as Brazil. Furthermore, it was suggested that the lack of an effect on BMI despite the major reduction in SSB and cookie consumption may be the result of a concurrent increase in fruit consumption.

The web-based computer-tailored intervention (FATaintPHAT) described by Ezendam *et al*. (2012) included a focus on reducing the consumption of SSBs and high-energy snacks. The objective of the intervention was to prevent excessive weight gain among adolescents aged 12–13 years through the delivery of eight 15-min modules over a 10-week school term. Although the intervention was associated with 0.54 lower odds of a high daily intake (>400 mL day^−1^) of SSBs compared to the control group at the end of the intervention (4 months), there was no significant difference at 24-month follow-up and no difference in anthropometric measures. The students perceived to be at greater risk, as determined by their baseline behaviours, did appear to benefit from the intervention to a greater extent (1.5 BMI change compared to 3.7 in the control group) at 24-month follow-up but the limitations of the study were the marked differences in the student characteristics at baseline.

### School-based educational programmes combined with environmental change

The large study by Muckelbauer *et al*. (2009) introduced environmental and educational interventions into schools to promote the increased water consumption amongst primary school-aged children in socially deprived areas of two German cities. It combined the provision of water fountains and water bottles with educational sessions on water delivered by teachers as part of the school curriculum. This intervention did not have a significant effect on soft drink or juice consumption but significantly increased water consumption (1.1 glasses day^–1^ or more; 95% CI = 0.7–1.4 glasses day^–1^; *P* < 0.001) compared to the control group. Although the change in BMI SD (i.e. standard deviation) score did not differ between the intervention and control group, the risk of being overweight was reduced by 31% (*P* = 0.04) in the intervention group. Measurement of the water flow of the drinking fountains during the intervention indicated that issuing new water bottles during the intervention led to increased water flow, suggesting that bottles may be an effective incentive in primary school-aged children. One limitation of this particular study is that other changes in dietary behaviours possibly resulting from this intervention were not recorded. Within the current review, this was the only one of the final selection of studies that attempted to estimate the costs of implementing the intervention. The costs associated with the environmental changes needed for this intervention were estimated as €2500 per water fountain and annual costs per child of €13 with no added costs for the educational component as it was delivered by the teachers.

### School-delivered drinks

The school-based intervention (DRINK) by de Ruyter *et al*. (2012) involved the masked replacement of SSBs with non-energy drinks for an 18-month period in primary school-aged children. This double-blinded randomised controlled trial (RCT) provided participating children with one can each day of either a non-carbonated, non-energy, artificially sweetened drink or a 435 kJ (104 kcal), noncarbonated, sugar-containing alternative. After 18 months, the intervention group had significant reductions in BMI *Z*-score of −0.13 (*P* = 0.001), mean weight gain (significant mean difference of 1.01 kg; *P* < 0.001) and body fat measurements compared to the control group. One of the strengths of this trial was the measurement of urinary sucralose as an additional compliance marker from randomly selected children who completed the study. These measurements suggested a high degree of adherence amongst participants. A limitation of the study is that 26% of participants in the intervention group did not complete the study and, when their data were included in the analysis, the effect of the study beverage became smaller and was no longer significant. It was concluded that this was likely a result of those children returning to regularly having sugary drinks again once they had withdrawn from the trial.

### Home-delivered drinks

Ebbeling *et al*. (2012) delivered water or diet drinks to the homes of randomly assigned overweight and obese secondary-aged children over a 12-month period. After 12 months, consumption of SSBs by the intervention group was almost non-existent (88% reduction in servings/week; *P* < 0.001) and remained very low at 2 years (76% reduction; *P* < 0.001) despite no active intervention in the intervening 12 months. This trial was the only one selected that also reported the energy and sugar intake from SSBs. Both the energy and sugar intake decreased significantly in the intervention group compared to the control group and these significant differences still existed at the 2-year mark. With respect to BMI, the net intervention effect in the experimental group at the end of 12 months was a significant reduction (−0.57; *P* = 0.045), although this was not significantly different at 2 years (−0.30; *P* = 0.46).

### Quality assessment

Each of the selected papers were scored according to randomisation, the appropriateness of the method of randomisation, whether blinding was included in the methodology and the robustness of the process, and also whether those children who dropped out of the intervention/control were described and accounted for in the data analysis. Thus, the maximum number that could be scored was 5 (Table2).

**Table 2:** Quality assessment criteria used to assess the final eight intervention studies identified in the systematic review

	Randomisation	Method of randomisation described and appropriate	Blinding mentioned	Method of blinding described and appropriate	Withdrawal and dropout of subjects described	Total
Cunha *et al*. (2013)	1	0	0	0	1	2
De Ruyter *et al*. (2012)	1	1	1	1	1	5
Ebbeling *et al*. (2012)	1	1	1	1	1	5
Ezendam *et al*. (2012)	1	0	0	0	1	2
James *et al*. (2004, 2007)	1	1	1	1	1	5
Muckelbauer *et al*. (2009)	1	1	0	0	1	3
Sichieri *et al*. (2008)	1	1	0	0	1	3
Singh *et al*. (2009)	1	1	0	0	1	3

Total quality assessment score for which scores range between 1 and 5: with 1 being the lowest quality and 5 being the highest quality.

Using this scoring system, the study by Cunha *et al*. (2013) received the lowest score of 2 because, although participating schools were randomised to either intervention or control, the randomisation process did not allow for the significant differences in the baseline characteristics of the participating children. Similarly, the study by Ezendam *et al*. (2012) scored 2 for similar reasons with the intervention group consisting of more vocational schools, more boys and more non-Western students than the control group.

Three studies (Muckelbauer *et al*. (2009); Sichieri *et al*. (2008); Singh *et al*. (2009) scored 3. The studies by de Ruyter *et al*. (2012); Ebbeling *et al*. (2012) and James *et al*. (2004) had the highest possible quality score of 5. Although receiving high scores, these studies still have limitations as described individually.

## Discussion

The aim of this systematic review was to identify interventions that can help to reduce the consumption of SSBs in children resulting in changes in body fatness so that conclusions can be drawn about how future effective interventions may be designed and used by relevant health professionals to address the increasing prevalence of childhood obesity.

Eight studies met the inclusion criteria. Six interventions achieved significant reductions in SSB intake but this was not always sustained over a longer period of time.

The interventions can be considered under four headings: school-based educational programmes, school-based educational programme combined with environmental change, school-distributed drinks and home-delivered drinks.

### School-based educational programmes

Educational programmes delivered via the school curriculum focussing solely on consumption of drinks can be effective in reducing the intake of SSBs (James *et al*., 2004; Sichieri *et al*., 2008; Singh *et al*., 2009; Ezendam *et al*.,^2012^; Cunha *et al*., 2013) and can have an impact on percentage overweight or obese (James *et al*., 2004) and reduction in BMI amongst those who are overweight, particularly in girls (Sichieri *et al*., 2008; Singh *et al*., 2009) and those who were perceived to be at greater risk at baseline (Ezendam *et al*., 2012).

In the nutritional education programme delivered by James *et al*. (2004), although only a 25% reduction in carbonated drinks containing sugars was achieved at 12-month follow-up, there was a 7.7% difference in percentage overweight or obese, with those in the intervention group being less at risk. This effect, however, was not sustained at 36-month follow-up and, unfortunately, the level of consumption of SSBs at 3 years was not reported. In the other education-based programmes, the risk of being overweight/obesity was reduced following the web-based computer-tailored intervention reported by Ezendam *et al*. (2012) but only in those children perceived to be at a greater risk at baseline. It was predicted that those engaging in more ‘risky’ health behaviours at baseline might benefit more from the intervention and, indeed, this was the case. However, in contrast, although the nutrition education sessions delivered in Brazil (Cunha *et al*., 2013) did lead to differences in soda (*P* = 0.02) consumption between the intervention and control groups, this did not lead to any significant differences in levels of body fatness. These children were from a relatively poor area of Brazil and it is proposed that the reduced intake of SSBs may have led to alternative sugar-containing beverages being consumed, which may not have been included in the analysis, or more fruit, which did not lead to a displacement of other less healthy items, as has been found in a number of systematic reviews of fruit intake and obesity (Rolls *et al*., 2004; Ledoux *et al*., 2011). A poor level of physical activity was also noted as a result of poor access to and inadequate facilities.

Generally, it is apparent that, although differences are seen for a certain length of time, either when the intervention is ongoing or after the intervention, behaviours are no different after a longer time interval.

Although not meeting the reviews inclusion criteria, Lo *et al*. (2008) demonstrated the effectiveness of the use of peers in changing behaviour in adolescents. The impact of peer leaders in school-based nutrition interventions in adolescents has been reported previously (Story *et al*., 2002). In the study by Lo *et al*. (2008), both the single and multiple peer groups maintained a decrease in SSB consumption at 3-month follow-up but returned to their baseline behaviour at 1 year. This return to baseline behaviour has been found in other studies of teenagers (Lytle *et al*., 2004) and it was suggested that this supports the potential need for maintenance sessions to remind children to maintain any new behaviours.

An evaluation of the multi-component Portuguese study (Program Obesity Zero), estimated to cost €373 per child, carried out by Rito *et al*. (2013) included a combination of health centre-based, family-centred and school-based education activities amongst overweight and obese primary school-aged children of low socioeconomic status. Following the 6-month intervention, SSB consumption was reduced by 78% from baseline. BMI was 0.4 kg m^–2^ (*P* < 0.001), which is significantly lower after the intervention with a waist circumference of ≤2 cm (*P* < 0.001), whereas vigorous physical activity had increased, screen viewing time decreased and intake of total energy was less. However, the study data are not reported in such a way as to make it possible to identify the impact of the reduced consumption of SSBs on the anthropometric measurements (BMI and waist circumference). The study limitations include the fact that 55.1% of families with overweight children chose nonparticipation and it is possible that children may have biased their 24-h recalls of dietary intake following the intervention. These limitations will be equally applicable to some of the studies included in the review.

### School-based educational programme combined with environmental change

The only study of this type from the final studies selected is Muckelbauer *et al*. (2009). The increased water consumption found in this trial in two poorer areas in Germany mirrors that found in a similar intervention amongst secondary school-aged children undertaken by Loughridge & Barratt (2005) and reinforces the impact of modifying the environment to support behavioural changes (Summerbell *et al*., 2005; Sharma, 2007). Environmental changes to schools and communities may well play a key role in altering SSB intake in children. Cradock *et al*. (2011) undertook a quasi-experimental evaluation of consumption data before and after policy changes regarding the sale of SSBs in vending and a la carte settings in US high schools and found that a significant reduction in SSB consumption coincided with a policy change to restrict their sale in high schools. A study by Kubik *et al*. (2011) highlighted the potential for school and district wellness councils to impact on the availability of less healthy vending machine fare in schools.

### School-distributed drinks

The study by de Ruyter *et al*. (2012) demonstrates the impact of providing non-energy drinks to children in the school environment. This high quality, rigorously conducted study overcomes many of the shortcomings of previous RCTs and provides strong evidence that the reduced consumption of SSBs can decrease weight gain and obesity in children. As highlighted by the study, the evidence from observational studies suggests that consuming artificially sweetened drinks is associated with weight gain not weight loss (Fowler *et al*., 2008; Foreyt *et al*., 2012). However, the findings of this randomised control intervention study would support a move to discourage children from consuming sugary drinks as one potential way of reducing the high incidence of overweight in children. It may offer a workable intervention that can be replicated cost effectively in school settings.

### Home-delivered drinks

The only study involving the home delivery of drinks to children is reported by Ebbeling *et al*. (2012). The study was designed to demonstrate the effects of reducing SSB consumption on body weight rather than as an intervention that could feasibly be rolled out on a wider scale. It was suggest that the lack of effect on BMI at the 2-year mark could either be the result of an increase in energy intake from SSBs and fruit juices in the intervention group once the trial has discontinued, a decrease in intake in the control group, or a combination of both.

### Relevance of research to practice and the public health agenda

The final studies selected in this review suggest that a medium intensity (between 4 and 10 × 1-h sessions delivered over a period ranging between 6 weeks and 12 months) nutrition education programme focussing on beverage choices and delivered by either peers, teachers or nutritionists could be an effective way of reducing consumption of sugary drinks in both primary and secondary school-aged children.

The use of computer or web-based nutrition education delivered via school and home may also offer an effective contemporary educational route in reducing SSB consumption in children. Ezendam *et al*. (2012) showed a reduced risk of high SSB consumption following a web-based secondary school intervention. Haerens *et al*. (2007) improved the fat intake of secondary school-aged girls using a computer-tailored intervention aimed at promoting healthy food, drink and physical activity choices. Computer-based interventions could be an effective tool in helping to reduce SSB consumption amongst this age group and possibly younger children too, although it must be acknowledged that computer-based interventions may not be appropriate for all international communities, particulary in developing countries in areas of socioeconomic deprivation.

A consistent strand running through a number of the final studies highlighted in this review is the suggestion that maintenance sessions are necessary to remind children to maintain any change in SSB consumption over time (James *et al*., 2004, 2007; Singh *et al*., 2009; Ebbeling *et al*., 2012; Ezendam *et al*.,^2012^). The need for maintenance sessions to be factored into the development of future interventions aimed at reducing SSB consumption is likely to be key to their long-term effectiveness.

Interventions that encourage possible alternative drinks to SSBs for children (water, diet drinks, 100% fruit juice) can offer some effective and practical ideas for future dietetic practice. The naturally occurring sugars in 100% fruit juices do mean that they contain a relatively high amount of energy (albeit along with some vitamins and other nutrients) and may be associated with an increased risk of excess weight gain (Dennison *et al*., 1997) particularly amongst children who are already overweight (Faith *et al*., 2006). Consumption should therefore be moderate. For example, the UK Eat Well Plate guidelines suggest 150 mL day^−1^ of fruit juice counts as one portion of the daily recommendation to have at least five fruits and vegetables (Public Health England, 2013). Fruit juice should only count towards one portion of this daily five-a-day recommendation of fruit and vegetables. In the USA, one recommended strategy for preventing childhood obesity is to ‘Increase access to free, safe drinking water in public places to encourage water consumption instead of SSBs’ (Institute of Medicine of the National Academies, 2009).

The combination of an educational intervention allied with changes to a primary school environment (provision of water fountains and water bottles) in the study carried out by Muckelbauer *et al*. (2009) did not lead to significantly reduced consumption of SSBs but did significantly increase water consumption and the risk of being overweight was reduced in the intervention group. The school-based educational study by Siega-Riz *et al*. (2011) aimed at improving a range of energy related behaviours also had no effect on SSB consumption but did significantly increase water consumption amongst secondary school-aged children.

Regarding switching from sugary drinks to plain water, it is worth noting that this may be potentially too large a leap for some children (Sichieri *et al*., 2008) and consideration should be given to the possible merit of switching to a low-sugar alternative instead.

The studies by Ebbeling *et al*. (2012) and de Ruyter *et al*. (2012) both involve providing either water or diet drinks to children as an alternative to SSBs. Both interventions resulted in significant reductions in SSB intake and significant reductions in BMI in the intervention groups. However, these studies were designed to demonstrate the effects of reducing SSB consumption on body weight rather than as interventions that could feasibly be rolled out on a wider scale. Ebbeling *et al*. (2012) suggest that further work is needed to develop practical interventions for reducing SSB consumption that can be implemented on a large scale, possibly involving altering the school and community environments (availability, pricing and marketing of SSBs and non-SSBs).

These studies highlight the potential role that health professionals can play in working with schools to ensure that school environments encourage children to make healthier drink choices. Hu *et al*. (2013) suggest that public policies can play a key role in changing SSB consumption amongst children. Policies that could be considered include public health campaigns, higher taxation on SSBs and restricting access to SSBs, especially large serving sizes.

It is worth noting that a number of studies were effective amongst children in low socioeconomic groups and therefore could offer an opportunity for helping to reduce current health inequalities amongst children. The study by Muckelbauer *et al*. (2009) prevented the prevalence of being overweight amongst primary school-aged children in socially deprived areas and the intervention delivered by Ezendam *et al*. (2012) produced a reduced increase in BMI in the intervention group over the 2-year follow-up period for those perceived to be at greatest risk of unhealthy behaviours. This benefit was seen despite the intervention group consisting of more vocational schools and vocational-level students than the control group.

One possible way forward may be to adopt a modular approach to developing interventions by initially focusing on reducing SSB consumption followed by efforts to then alter other energy balance related behaviours in turn. This would give participants the opportunity to focus their efforts on one element at a time, without having to make too many stretching lifestyle changes at once. The maintenance of newly learned behaviours could also be built into the latter stages of an intervention to reinforce them. This may be a future area of research worth pursuing.

### Strengths and limitations

Strict inclusion criteria was identified in advance of the search process and used accurately to identify relevant studies. The studies were quality assessed against a validated quality assessment tool designed to assess the quality of RCTs (Jadad *et al*., 1996).

One limitation of this review is the small number of studies selected for comparison. This highlights the current small available pool of evidence in this area from which to draw meaningful conclusions about effective future interventions. The final studies are heterogeneous in nature and any overall conclusions must be drawn with care. Additionally, unpublished data and studies were not included within this review.

No studies were identified where an intervention targeted children under the age of 8 years.

## Conclusions

The number of RCTs undertaken to date that are designed to reduce the consumption of SSBs and reduce body fatness in children is limited. No studies have been published to date that investigate interventions aimed at preschool-aged children. Based on evidence from the studies that have been completed in this field, school-based education programmes focused on reducing SSB consumption and incorporating follow-up modules may offer health professionals the best opportunities for implementing effective and sustainable interventions that are effective in both children and adolescents. Changing the school environment to support such educational programmes could improve the effectiveness of these interventions. There is a lack of relevant reported interventions carried out outside of the school environment. It should be noted that the school-based evidence does, however, include certain aspects that may be reproducible and effective in other settings. More rigorously conducted, quality RCTs in this area are required to aid the design of effective interventions that can be implemented by health professionals as one strand in the strategy of addressing the childhood obesity epidemic.
